# Sequential intranasal booster triggers class switching from intramuscularly primed IgG to mucosal IgA against SARS-CoV-2

**DOI:** 10.1172/JCI175233

**Published:** 2025-01-14

**Authors:** Yifan Lin, Xuejiao Liao, Xuezhi Cao, Zhaoyong Zhang, Xiuye Wang, Xiaomeng He, Huiping Liao, Bin Ju, Furong Qi, Hairong Xu, Zhenhua Ren, Yanqun Wang, Zhenxiang Hu, Jiaming Yang, Yang-Xin Fu, Jincun Zhao, Zheng Zhang, Hua Peng

**Affiliations:** 1 Department of Basic Medical Sciences, School of Medicine, Tsinghua University, Beijing, China.; 2 Institute of Biophysics, Chinese Academy of Sciences, Beijing, China.; 3 Institute for Hepatology, National Clinical Research Center for Infectious Disease, Shenzhen Third People’s Hospital, The Second Affiliated Hospital, School of Medicine, Southern University of Science and Technology, Shenzhen, China.; 4 Guangzhou Laboratory, Guangzhou International Bio-Island, Guangzhou, China.; 5 State Key Laboratory of Respiratory Disease, National Clinical Research Center for Respiratory Disease, Guangzhou Institute of Respiratory Health, the First Affiliated Hospital of Guangzhou Medical University, Guangzhou, Guangdong, China.; 6 Changping Laboratory, Beijing, China.; 7 LivzonBio Inc., Zhuhai, Guangdong, China.

**Keywords:** COVID-19, Immunology, Adaptive immunity, Cytokines

## Abstract

The persistent emergence of COVID-19 variants and recurrent waves of infection worldwide underscores the urgent need for vaccines that effectively reduce viral transmission and prevent infections. Current intramuscular (IM) COVID-19 vaccines inadequately protect the upper respiratory mucosa. In response, we have developed a nonadjuvanted, IFN-armed SARS-CoV-2 fusion protein vaccine with IM priming and intranasal (IN) boost sequential immunization. Our study showed that this sequential vaccination strategy of the IM+IN significantly enhanced both upper respiratory and systemic antiviral immunity in a mouse model, characterized by the rapid increase in systemic and mucosal T and B cell responses, particularly the mucosal IgA antibody response. The IN boost triggered a swift secondary immune response, rapidly inducing antigen-specific IgA^+^ B cells. Further B cell receptor–seq (BCR-seq) analysis indicated that these IgA^+^ B cells primarily arose through direct class switching from preexisting IgG^+^ B cells in draining lymph nodes. Notably, our clinical studies revealed that the IN boost after IM vaccination elicited a robust systemic IgA antibody response in humans, as measured in serum. Thus, we believe that our cytokine-armed protein vaccine presents a promising strategy for inducing rapid and potent mucosal protection against respiratory viral infections.

## Abstract 2

The intranasal vaccine booster after intramuscular prime enhances mucosal immunity against SARS-CoV-2 by promoting IgG+ to IgA+ B cell class switching.

## Graphical abstract

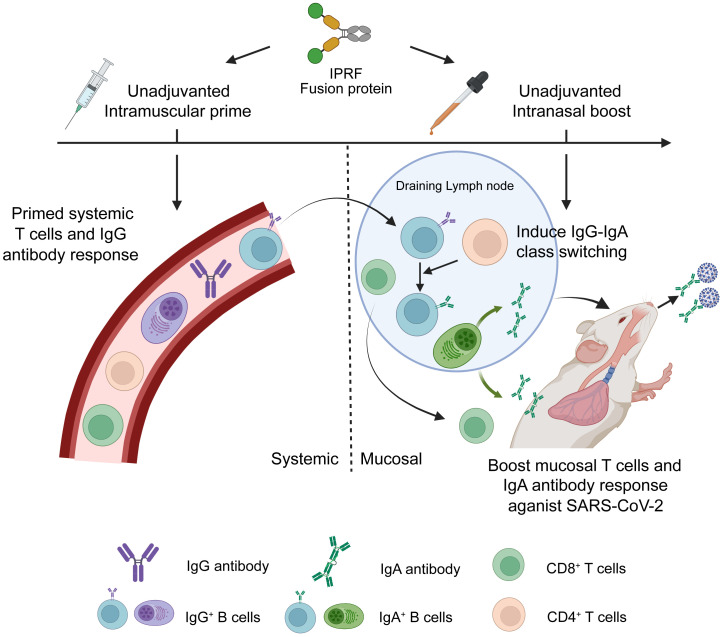

## Introduction

COVID-19 presents substantial health challenges, with emerging variants leading to recurrent waves of infection worldwide (1). Currently, viral vaccines, including inactivated, adenovirus-packaged, and mRNA formulations, are predominantly administered via intramuscular (IM) injection to stimulate systemic antiviral immunity (2–5). These vaccines have played essential roles in reducing severe illness and mortality in COVID-19 (6). However, the parenteral vaccines have notable limitations in generating mucosal immunity in the upper respiratory tract, leaving behind inadequate effectiveness in curbing viral transmission and spread (7). This deficiency creates a substantial reservoir of infected individuals, facilitating ongoing viral persistence and replication. Consequently, persistent cycles of replication and infection may expedite the emergence of viral mutations (8, 9), enabling the virus to evade immune responses triggered by vaccination or prior infection and culminating in reinfection (10, 11).

Mucosal vaccines present marked advantages for controlling viral infection of upper respiratory tract because they induce secretory IgA antibodies within the mucosa environment (12). High-affinity IgA provides a frontline of immune defense at mucosal surfaces, blocking and neutralizing toxins and pathogenic microbes (13, 14). During the early stages of SARS-CoV-2 infection, the IgA response predominates in the neutralizing antibody response within the respiratory tract, facilitating viral clearance (15, 16). Moreover, the incidence of breakthrough infections inversely correlates with the strength of the IgA antibody response (17). Thus, the IgA response is crucial for assessing protective immunity against SARS-CoV-2 and evaluating vaccine efficacy.

However, mucosal vaccine development is limited by the lack of adjuvants that can effectively and safely enhance nasal mucosal immune responses (18). Existing adjuvants formulated for IM administration are not approved by the FDA for nasal delivery, posing a challenge in inducing mucosal immunity by protein vaccines (19). Our previous study reported on a fusion protein vaccine containing the receptor-binding domain (RBD) of the SARS-CoV-2 spike protein and an interferon, IFNα-Pan-RBD-Fc (IPRF). This IPRF fusion protein, as an IM vaccine, can induce potent antiviral immune responses without the need for exogenous adjuvants (20). The human-adapted formulation of this vaccine, named V-01, as an IM vaccine, has successfully undergone 3 phases of clinical studies, demonstrating a robust neutralizing antibody response and excellent safety profiles in both adult and even elderly groups (21). Importantly, this IPRF fusion protein vaccine is also suitable for nasal administration, which can elicit a robust mucosal IgA and T-cell response against SARS-CoV-2 (22).

To explore potential strategies for future vaccine booster immunizations, it is imperative to ascertain whether intranasal (IN) boosters can effectively elicit robust mucosal immune responses following IM vaccine priming. In this study, we investigated whether and how IPRF, as an IN booster following IM prime, can rapidly induce mucosal and systemic T and B cell response, especially mucosal IgA response, and completely protect mice from SARS-COV-2 infection. Mechanistically, the rapidly increased antigen-specific IgA is attributed to the secondary class switching of antigen-specific IgG^+^ B cells primed intramuscularly in a CD4^+^T cell–dependent manner.

## Results

### Only IN but not IM sequential booster induces an RBD-specific IgA immune response.

**Figure 1:**
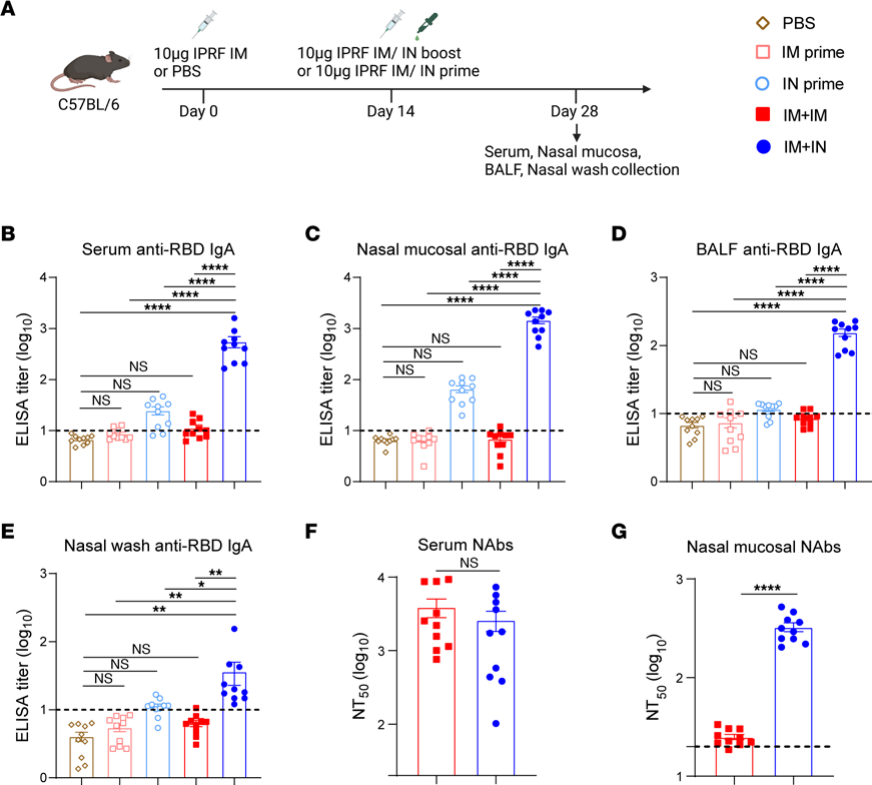
Only IN but not IM sequential immunization induces RBD-specific IgA mucosal immune response. (**A**) C57BL/6J mice (6–8 weeks, *n* = 10) were immunized intramuscularly with 10 μg IPRF or PBS, followed by a boost with 10 μg IPRF either intramuscularly or intranasally 14 days later. (**B**–**E**) The RBD-specific IgA antibody responses in serum, nasal mucosa, BALF, and nasal washes of immunized mice were evaluated 28 days after priming by ELISA. The dotted lines represent the endpoint of these ELISA tests. (**F** and **G**) The neutralization activity of vaccinated sera and nasal mucosa collected on day 28 was evaluated using a pseudovirus neutralization assay. The dotted lines represent the minimum dilution. *P* values were determined by 1-way ANOVA with Tukey’s multiple comparisons test. *P < 0.05, **P < 0.01, ****P < 0.0001. Individual data points are represented and were pooled from 2 independent experiments.

Our previous study demonstrated that nasal delivery of the IPRF vaccine induces potent antiviral immune responses without the need for exogenous adjuvants (20). We sought to investigate whether boosting intramuscularly primed mice with the IPRF fusion protein vaccine could foster both mucosal and systemic immunity. We vaccinated C57BL/6J mice with IPRF or PBS via IM injection, followed by either IM (IM+IM) or IN sequential (IM+IN) administration of the same dose of IPRF without additional adjuvants 14 days later. The PBS-primed mice boosted with IPRF via IM or IN served as IM prime or IN prime controls. Mice were euthanized on day 28 (Figure 1A), and mouse sera, nasal mucosa supernatants, bronchoalveolar lavage fluid (BALF), and nasal wash were collected to examine anti-SARS-CoV-2 RBD IgG and IgA antibody responses. Compared with IM or IN priming alone, both IM+IM and IM+IN vaccinations elicited robust anti-RBD IgG antibody responses in serum and BALF (Supplemental Figure 1, A and B; supplemental material available online with this article; https://doi.org/10.1172/JCI175233DS1). Both strategies induced high titers of neutralizing antibodies in serum (Figure 1F), implying that both boost procedures can stimulate strong systemic immune responses. However, IM+IM resulted in a limited RBD-specific IgA antibody response in serum (Figure 1B) and nearly undetectable mucosal IgA levels (Figure 1, C–E). In contrast, the IM+IN approach led to high levels of both systemic and mucosal anti-RBD IgA, with neither IM prime nor IN prime alone sufficient for mucosal antibody development (Figure 1, B–E). Notably, the IM+IN strategy, but not the IM+IM, induced substantial levels of neutralizing antibodies in the nasal mucosa (Figure 1G). These findings confirm that an IN booster can effectively stimulate strong mucosal and systemic IgA responses following the initial IM vaccination.

**Figure 2:**
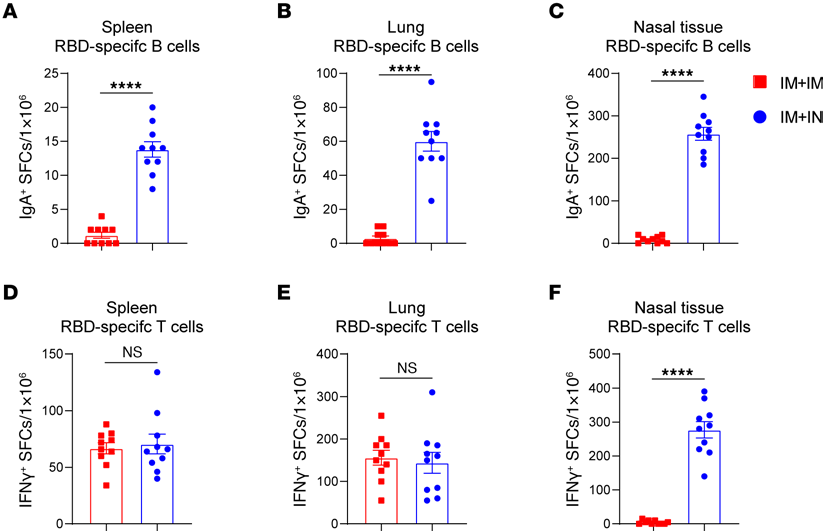
IN sequential immunization induces both systemic and mucosal RBD-specific IgA^+^ B cell and T cell responses. C57BL/6J mice (*n* = 10) were primed with 10 μg IPRF intramuscularly and boosted via IM or IN routes. Spleen, lung tissue, and NALT were collected on day 14 after boost. (**A**–**C**) ELISPOT assays were performed to measure IFN-γ secretion from splenocytes, NALT, and lung lymphocytes stimulated with an RBD peptide pool. (**D**–**F**) ELISPOT assay assessed IgA^+^ B cells from splenocytes, NALT, and lung lymphocytes cocultured with RBD protein. The data are presented as mean ± SEM. *P* values were determined by 1-way ANOVA with Tukey’s multiple comparisons test. ****P < 0.0001. Individual data points are represented and were pooled from 2 independent experiments.

Tissue-resident B cells generate more potent and rapid defensive responses than circulating B cells (23), particularly regarding the IgA response in the upper respiratory tract, which is critical for blocking viral infection (24). To determine whether IM+IN vaccination could induce memory B cell immune responses in mouse mucosa, we collected spleens from mice and evaluated antigen-specific IgG^+^ and IgA^+^ antibody-secreting cells (ASCs) using ELISPOTS analysis. Consistent with the antibody results, both the IM+IM and IM+IN groups exhibited strong antigen-specific IgG^+^ B cell responses in the spleen and lungs (Supplemental Figure 2, A and B). However, only IM+IN but not IM+IM vaccination elicited robust systemic and mucosal antigen-specific IgA^+^ B cell responses (Figure 2, A–C). To further characterize the subsets of the IM+IN–induced antigen-specific B cells, intracellular staining was performed for IgA^+^ lymphocytes from spleen, lung, and nasal tissue after incubation with RBD protein (Supplemental Figure 9A). We observed increased levels of memory IgA^+^ B cells (B220^+^ IgD^−^IgM^−^CD38^+^) (Supplemental Figure 3, A–D) and IgA^+^ ASCs (IgD^−^IgM^−^CD138^+^) (Supplemental Figure 3, E–H) in these tissues. Collectively, these data suggest that IN booster following IM priming can effectively elicit robust mucosal B cell responses.

### IN sequential immunization induces systemic and mucosal T cell responses.

Based on the antibody responses observed, we hypothesized that the IN booster could effectively recruit the systemic T cells generated by previous IM vaccination to the upper respiratory tract and lungs. To test this hypothesis, we analyzed antigen-specific T cell activation of lymphocytes isolated from the spleens, lungs, and nasal tissues of immunized mice. Both IM+IM and IM+IN immunization significantly increased antigen-specific T cells in the spleen and lungs (Figure 2, D and E). However, only the IM+IN group exhibited pronounced antigen-specific T cell activation in the nasal cavity (Figure 2F), suggesting that the IN booster, rather than the IM booster, induces a more potent T cell immune response in the upper respiratory tract.

### The IN sequential immunization protects K18-hACE2 transgenic mice against the SARS-CoV-2 challenge.

**Figure 3:**
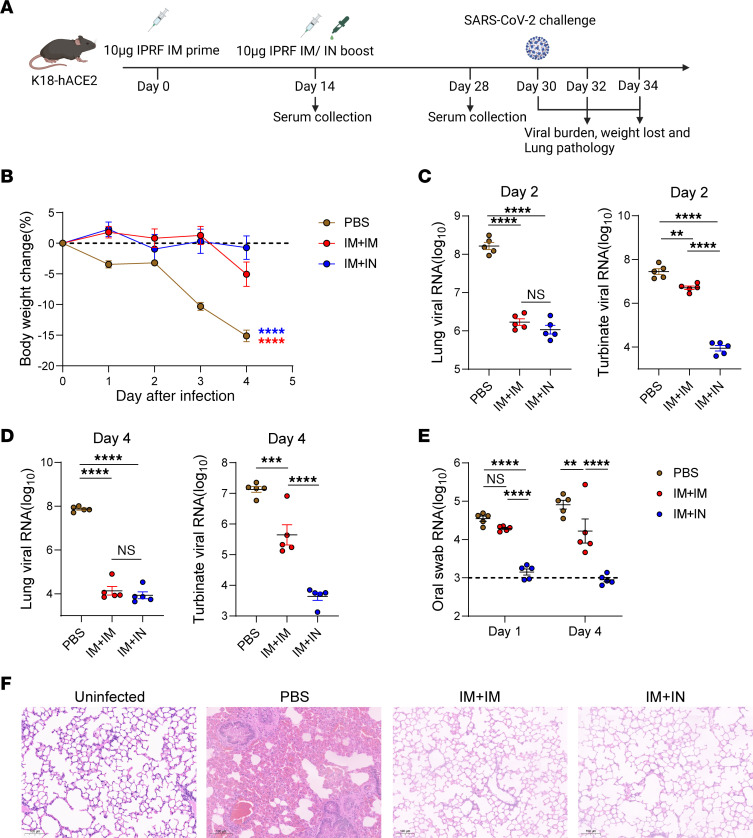
IN sequential immunization protects against COVID-19–like disease. (**A**) K18-hACE2 transgene mice (*n* = 10) were IM immunized with 10 μg IPRF or PBS on days 0 and boosted with 10 μg IPRF via IM or IN Mice were challenged with SARS-CoV-2 (Wuhan/WIV04/2019) on day 16 after boost. Five mice from each group were euthanized 2 days after challenge, while for the remaining 5 mice in each group, oral swabs were collected on days 1 and 4, then mice were euthanized 4 days after challenge. The lung tissues were collected for histological assessment 4 days after infection. Viral RNA copies in the lung and nasal turbinate tissue of each mouse were determined by qRT-PCR and plotted as log_10_ copies per mL. (**B**) Weight loss of PBS, IM+IM, or IM+IN mice was recorded from days 1-to-4 after infection. The dotted lines represent the no change of weight. (**C** and **D**) Infectious virus titers in lung and nasal turbinate tissues were measured on days 2 and 4 after infection. (**E**) Viral titers from oropharyngeal swabs were assessed on day 1 and 4 after infection. The dotted lines represent the measurement values obtained from the ddH_2_O wells. (**F**) Representative H&E staining results from uninfected, PBS, IM+IM, or IM+IN mice. Scale bar: 100μm. The data are presented as mean ± SEM. *P* values were determined by 1-way ANOVA with Tukey’s multiple comparisons test. **P < 0.01, ***P < 0.001, ****P < 0.0001.

In previous studies, we evaluated the protection efficacy of the IPRF vaccine against SARS-CoV-2 through 2 doses delivered either intramuscularly or intranasally. The IN IPRF vaccine demonstrated more efficient viral clearance in the upper respiratory tract than the IM IPRF vaccine (20, 22). To determine whether the IN boost in IM-primed mice would provide an effective protective enhancement, K18-hACE2 mice were primed with IPRF or PBS via IM, followed by IM or IN boosting with IPRF, consistent with prior experiments. After immunization via IM+IM or IM+IN, robust serum IgG and IgA responses, as well as high titers of SARS-CoV-2 neutralization antibodies, can be detected in K18-hACE2 mice (Supplemental Figure 4, A and B). Sixteen days after the boost, all groups of mice were challenged with SARS-CoV-2 and assessed for viral burden of nasal turbinate and lung on days 2 and 4 after infection. Lung pathology was assessed 4 days after infection. Mouse weight was monitored throughout the experiment. Oral swabs were collected on days 1 and 4 after infection (Figure 3A). Mice receiving IM+IM or IM+IN showed complete protection from weight loss, unlike the PBS control group (Figure 3B). While both IM+IM and IM+IN vaccination reduced lung viral burden and mitigated lung pathology, only the IM+IN vaccination effectively decreased the viral loads in both the upper respiratory tract (nasal turbinate and oral swab) and lower respiratory tract (lungs) (Figure 3, C–F). Thus, IN-sequential immunization emerged as a robust, safe, and protective vaccination strategy.

### An IN booster induces a rapid and robust secondary immune response upon IM priming.

**Figure 4:**
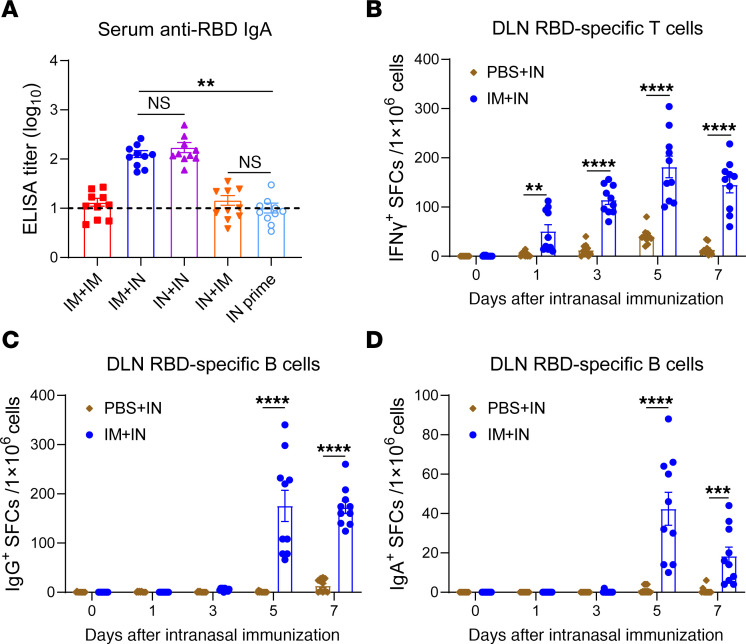
An IN booster induces a rapid and robust secondary immune response upon IM priming. (**A**) C57BL/6J mice (*n* = 10) were primed with 10 μg IPRF and boosted via IM or IN routes and subsequently boosted via either route. RBD-specific IgA antibody responses in sera were evaluated 7 days after boost by ELISA. The dotted lines represent the endpoint of these ELISA tests. (**B**–**D**) C57BL/6J mice were immunized with 10 μg IPRF or PBS via IM on day 0, followed by IN immunization with 10 μg IPRF on day 14 after prime. DLNs were harvested on days 0, 1, 3, 5, and 7 (each timepoint *n* = 10). ELISPOT assays assessed T cells, IgG^+^ or IgA^+^ B cells from mice DLN lymphocytes. The data are shown as mean ± SEM. *P* values were determined by 1-way ANOVA with Tukey’s multiple comparisons test. **P < 0.01, ****P < 0.0001. Individual data points are represented and were pooled from 2 or 3 independent experiments.

To compare the IgA responses induced by various vaccination procedures, we immunized mice with the following prime and boost combinations: IM+IM, IM+IN, IN+IN, IN+IM, or IN prime alone. Serum anti-RBD IgG and IgA response was examined at an early stage, specifically within 7 days after boost. All prime-then-boost immunization protocols elicited comparable serum IgG responses (Supplemental Figure 5A). Notably, the IM+IN group generated serum IgA levels equivalent to those produced by IN+IN, both of which were significantly higher than the levels induced by IN priming alone (Figure 4A). Conversely, the IM boost did not further enhance the antigen-specific IgA response, highlighting that only the IN boost could provoke a substantially stronger IgA immune response.

Secondary immune responses induced by an in situ boost vaccination are well documented to be both faster and more robust (25, 26). We proposed that IN booster following IM immunization could elicit a secondary immune response. To compare the time kinetics of immune responses induced by IM+IN versus single IN vaccination, we intranasally vaccinated both IM-primed and unvaccinated mice with IPRF. Mandibular draining lymph nodes (DLNs) were collected from mice on days 0, 1, 3, 5, and 7 after the boost to assess antigen-specific T and B cell responses. IM+IN vaccination resulted in a striking increase in antigen-specific T cells within the DLNs by day 1, indicating that IN administration following IM vaccination rapidly triggered a secondary T cell immune response in the upper respiratory tract (Figure 4B). By 3 days after vaccination, IgG^+^ B cell responses were detectable in DLNs in the IM+IN mice, whereas the single-IN group exhibited a delayed and weak IgG immune response peaking around day 7 after vaccination (Figure 4C). Antigen-specific IgA responses emerged by day 5, but nearly no IgA^+^ ASCs were observed in the DLNs of IN-primed mice (Figure 4D). These findings suggest that the IN booster effectively induced secondary IgA responses following IM priming.

### RBD-specific IgA^+^ B cells exhibit high clonal similarity to IgG^+^ B cells.

In the context of IM+IN immunization, 3 potential pathways can be postulated: (a) the classical IgM-IgA class-switch pathway (13, 14); (b) the induction of proliferation of a small preexisting population of IgA^+^ B cells; and (c) direct antigen-specific IgG-IgA class switching. The prompt induction of the IgA response suggests that it reflects a secondary response, making the first assumption less likely to be the primary mechanism. Following the IN boost, IgA^+^ ASCs were detected significantly later than IgG^+^ ASCs in DLNs, indicating that the induced IgA response is not derived from preexisting IgA^+^ B cells (Figure 4). Furthermore, repeated IM vaccinations do not expand IgA responses, suggesting that the second possibility is also unlikely.

**Figure 5:**
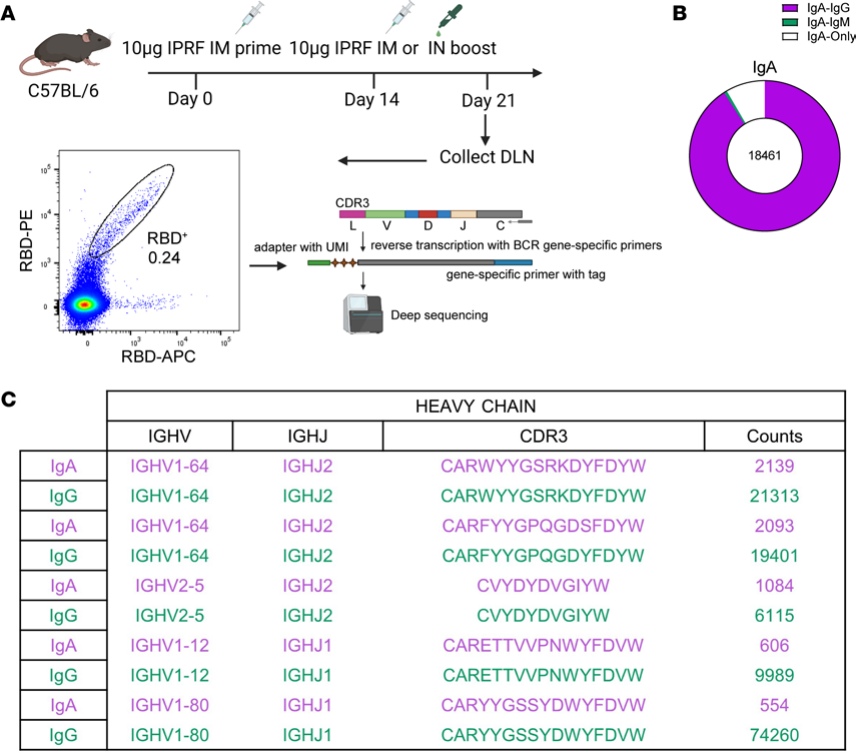
RBD-specific IgA^+^ B cells exhibit high clonal similarity to IgG^+^ B cells. (**A**) C57BL/6J mice (*n* = 20) were primed with 10 μg IPRF via IM+IN or IM+IM immunization. RBD-specific B cells were collected by flow sorting from mandibular lymph nodes (IM+IN) or inguinal lymph nodes (IM+IM) 7 days after boost. The total RNAs of those B cells were extracted for UMI BCR-seq. (**B**) The ratio of the identical clone type of IgA is shown in a pie chart. (**C**) Sample IgH sequence alignment for antibodies of IgG or IgA isotypes reveals identical IGH V(D)J and IGL VJ genes with highly similar CDR3 regions.

To characterize the antigen-specific IgA antibodies elicited by IM+IN immunization, we sorted antigen-specific B cells from the DLNs on day 7 after IM+IM or IM+IN immunization and performed B cell receptor–seq (BCR-seq) with unique molecular identifiers (UMIs) (Figure 5A and Supplemental Figure 9B). After removing clone reads below 10, we obtained 542,278 and 641,968 Ig heavy chain (IGH) sequences from the IM+IM and IM+IN groups, respectively. As expected, these antigen-specific IGH sequences in both groups were predominantly IgG, with IgA sequences detectable only in the IM+IN group (Supplemental Figure 6A). In the IM+IN group, the CDR3 length distribution of IgA mirrored that of IgG, demonstrating a synchronized peak, while there was no CDR3 length distribution of IgA as expected in the IM+IM group (Supplemental Figure 6B). We subsequently assessed the pair usage of IGHV-IGHJ gene segments, observing comparable V-J gene usage of IgA and IgG within the IM+IN group (Supplemental Figure 6C). Clustering of BCR sequences based on identical VDJ combinations and CDR3 sequences revealed that 90.92% of IgA sequences were clustered with IgG sequences in the IM+IN group (Figure 5, B and C). The proportion of sequences exhibiting homology to IgA within IgG reaches 72.70% (Supplemental Figure 6D). Further analysis of IGHV gene mutation rates of the IM+IN group indicated that IgA exhibited the highest IGHV mutation rate among IGHV genes, suggesting more advanced mutations of IgA following IgG to IgA class switching (Supplemental Figure 6E). Those results strongly imply that the direct IgG-IgA class switching occurred immediately following the IN sequential boost.

### RBD-specific IgA^+^ B cells induced by IN booster primarily originate from IM-primed IgG^+^ B cells in a CD4^+^ T cell–dependent manner.

IgA responses develop through highly complementary T cell–independent and T cell–dependent pathways (27). To investigate whether IM+IN vaccination induces IgA in a T cell–independent manner, we depleted CD4^+^ T cells with antibodies in mice previously immunized intramuscularly with IPRF, followed by IN immunization of the IPRF vaccine (Supplemental Figure 7A). Notably, the IgA response in serum was completely abolished following CD4^+^ T cell depletion (Supplemental Figure 7B). These data suggest that the IgA antibody response triggered by IM+IN immunization is contingent upon the presence of CD4^+^ T cells.

To further investigate the origin of these antigen-specific IgA^+^ B cells, we first isolated splenocytes from mice that had received IM injections of the IPRF vaccine and transferred 1 × 10^7^ cells per mouse into Rag-1 mice. After transfer, we immunized the recipient mice via IM or IN with IPRF protein (Supplemental Figure 8A). The RBD-specific IgG antibody response was detected in the serum of Rag-1 mice after both IM and IN immunization (Supplemental Figure 8B). However, the IgA antibody response was solely observed in the IN boost group (Supplemental Figure 8C). This provides compelling evidence that the IgA response can only be induced by IN boosters in Rag-1 mice receiving IPRF-primed splenocytes.

**Figure 6:**
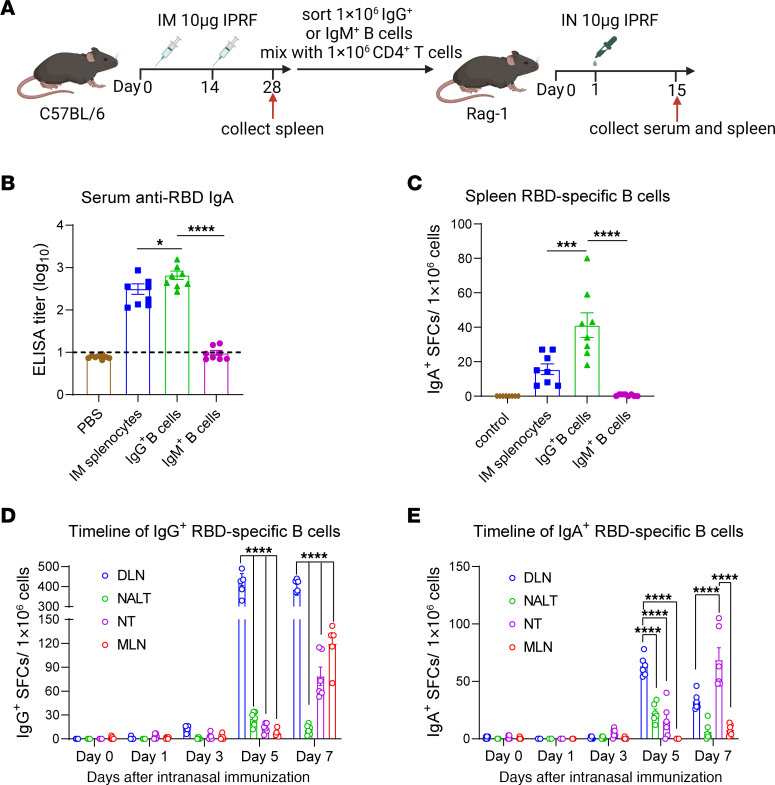
RBD-specific IgA^+^ B cells predominantly class switch from IgG^+^ B cells following IN sequential immunization. (**A**) Splenocytes from C57BL/6J (*n* = 10) mice receiving 2 doses of IM vaccination were collected and sorted for the IgG^+^ and IgM^+^ B cells and CD4^+^ T cells, which were then adoptively transferred into Rag-1 mice (*n* = 8). A day later, the mice were intranasally administrated 10 μg IPRF. The dotted lines represent the endpoint of these ELISA tests. (**B**) RBD-specific IgA antibody response in sera of immunized Rag-1 mice was evaluated 14 days after vaccination by ELISA. (**C**) ELISPOT assay measured IgA^+^ B cells from splenocytes cocultured with RBD protein. (**D** and **E**) C57BL/6J mice (*n* = 30) received 10 μg IPRF via IM at day 0, followed by IN immunization with 10 μg IPRF on day 14 after prime. Nasal draining mandibular lymph nodes (DLN), nasal-associated lymphoid tissue (NALT), nasal turbinate (NT), and mediastinal lymph node (MLN) were harvested on days 0, 1, 3, 5, and 7 (each timepoint, n=6), and lymphocytes were collected. ELISPOT assessed IgG^+^ (**D**) or IgA^+^ (**E**) B cells from these lymphocytes. The data are presented as mean ± SEM. *P* values were determined by 1-way ANOVA with Tukey’s multiple comparisons test. *P < 0.05, ***P < 0.001, ****P < 0.0001. Individual data points are represented and pooled from 2 independent experiments.

Given that IM priming predominantly induces IgG^+^ and IgM^+^ B cell responses, we assessed whether the antigen-specific IgA^+^ B cells were derived from IgM^+^ or IgG^+^ B cells. We sorted IgG^+^ B cells or IgM^+^ B cells from the spleens of intramuscularly vaccinated mice and transferred them, along with an equal number of CD4^+^ T cells, to Rag-1 mice (Supplemental Figure 9C). Control groups included no transfer and transfer of 1 × 10^7^ total splenocytes as negative and positive controls, respectively. A day after transfer, the mice received the IPRF vaccine intranasally (Figure 6A). In the IgG^+^ B cell–transferred group, a strong antigen-specific serum IgG and IgA response was observed (Supplemental Figure 8D and Figure 6B). Concurrently, antigen-specific IgG^+^ and IgA^+^ ASCs were also detected in the spleens of these mice (Supplemental Figure 8E and Figure 6C). In contrast, the IgM^+^ B cell transfer group showed no detectable IgA responses. This finding clearly indicates that the IgA response induced by IM+IN is directly derived from IgG^+^ B cells, rather than from IgM^+^ B cells.

Recent research has demonstrated that the IgA-secreting cells originate from nasal-associated lymphoid tissues (NALTs) following nasal vaccination and subsequently migrate to nasal turbinate (NT) (28). However, our study revealed a strong antigen-specific IgA ASC response in the submandibular DLNs. To determine the key anatomical site for antigen-specific IgG-IgA class switching during IM+IN immunization, we collected the DLNs, NALTs, NT, and mediastinal lymph node (MLN) from mice on days 0, 1, 3, 5, and 7 after boost to assess antigen-specific B cell responses. Consistent with the previous experiments (Figure 4C), the earliest detectable IgG response occurred in the DLN on day 3. Notably, on days 5 and 7, the DLN exhibited a significantly stronger IgG response compared with the other tissues (Figure 6D). The earliest IgA response was observed in the DLN on day 5, but, by day 7, the nasal cartilage showed strongest IgA response (Figure 6E). These results suggest that the DLN may serve as a critical site for antigen-specific IgG-IgA class switch during IM+IN immunization. Ultimately, these antigen-specific IgA cells may be home to the NT, thereby providing immune protection in the upper respiratory tract.

### IN sequential immunization is well tolerated and elicits enhanced mucosal immunity in humans.

We then conducted an investigator-initiated clinical trial to test the short-term safety and mucosal immune responses in volunteers age 28-to-50 years old who had already received 2 or 3 doses of the inactivated COVID-19 vaccine from Sinovac for 6 months. A total of 30 vaccinated volunteers were screened for eligibility between March 28 and April 16, 2022. Thirty participants (93.75%) were finally enrolled, of whom 5 (16.7%) entered the low-dose group in batch 1, and 25 (83.3%) entered the high-dose group in batch 2 (Supplemental Table 1). Notably, no severe adverse events were reported in any participants during the first 28 days after booster vaccination. The most common adverse reaction was dry nose symptoms occurring in both the low-dose group (1 [20%]) and high-dose group (4 [16%]), followed by swelling and nasal congestion in the low-dose cohort and sneezing and runny nose in the high-dose group. Importantly, no significant difference in the incidence of adverse reactions was observed between the 2 groups (Supplemental Table 2). These clinical data indicate that IN sequential immunization with an unadjuvanted IFN-armed fusion protein is well tolerated in humans.

**Figure 7:**
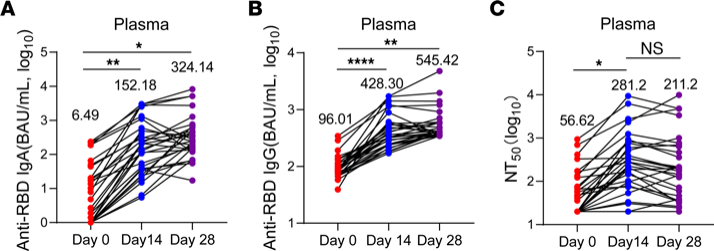
IN sequential immunization significantly enhances mucosal immunity in humans. All participants (*n* = 30) received a single dose of the V-01 vaccine via nasal spray, and the plasma samples were collected on the day of vaccination and on days 14 and 28 after IN boost. (**A** and **B**) The RBD-specific IgG and IgA antibody responses in plasma were evaluated by ELISA. (**C**) The neutralization activity of plasma was evaluated using a pseudovirus neutralization assay. Each data point represents an individual plasma sample. Data are presented as mean ± SEM. *P* values were determined by 1-way ANOVA Tukey’s multiple comparisons test. *P < 0.05, **P < 0.01, ****P < 0.0001.

We also assessed the capacity of this IN booster to enhance human IgG and IgA levels in the 30 volunteers recruited for the study. Prior to the nasal challenge, analysis of RBD-specific antibody response in sera revealed low serum IgG antibody levels and nearly undetectable IgA responses. However, 14 days following nasal spray administration of the V-01 vaccine, a remarkable increase in RBD-specific IgA was observed in plasma in most volunteers, accompanied by significantly increased anti-RBD IgG levels (Figure 7, A and B). At the same time, the neutralization titer in volunteer plasma also experienced a substantial increase (Figure 7C). These findings demonstrate that the IM+IN immunization strategy employing V-01 significantly augments the protective IgA and IgG in the serum of humans.

## Discussion

In this study, we performed IN booster vaccinations in mice using the IPRF vaccine following IM priming and confirmed that this adjuvant-free fusion protein can elicit robust systemic and upper respiratory antiviral immune responses. The IPRF booster provokes a robust antigen-specific IgA response in both the lungs and nasal mucosa, effectively enhancing mucosal protection in the upper respiratory tract, which is often insufficient following IM-only immunization. Further analysis revealed that the immune response induced by IPRF nasal sequential immunization was remarkably rapid, resembling secondary immunity. Additionally, the IM+IN vaccination strategy generates a significantly stronger viral antigen-specific mucosal T cell response than either IN priming or repeated IM immunization. Notably, we provide the first evidence of antigen-specific IgG-IgA class switch induced by IM+IN immunization in vivo, confirming that the mandibular DLNs are the critical site where this IgG-IgA class-switch occurs.

The COVID-19 pandemic has underscored the critical importance of mucosal vaccines (29). Currently, nearly all FDA-approved IN vaccines are viral based, because of their strong immunogenicity, adjuvant independence, and self-delivery capabilities (19, 30–32). However, the preexisting immune response to viral vectors can hinder the effectiveness of these vaccines, thereby limiting the potential for repeated immunization (33). Recombinant proteins with efficient nasal absorption, safe adjuvants, robust immunogenicity, and determined antiviral mechanisms hold great promise as mucosal vaccine candidates.

The substantial IgA response elicited by IM+IN vaccination in mice suggests that the IN booster amplifies preexisting immunity rather than initiating a primary IgA response. Similar findings indicate that a combination of IM prime and IN boosting provides enhanced protection against pathogens across various vaccine platforms (34–37). Studies on influenza reinfection have demonstrated that secondary IgA production can be augmented through IgA^−^ memory B cells (38). Furthermore, research into human antibody repertoires in autoimmune diseases and lineage tracing of human B cells suggests that IgA production may occur through both the classical class switch from IgM^+^ to IgA^+^ and conversion from the IgG^+^ subtype (39, 40). Notably, the termed circle transcripts (CT) of IgG-IgA have been identified in both human and murine B cells via in vitro experiments (41–43). These previous findings lend experimental and theoretical support to our results.

While our study primarily emphasizes the IPRF booster-induced class switching from IgG to IgA, it is crucial and intriguing to compare various vaccine formulations or antigen components to assess the generalizability of this class-switching phenomenon. We analyzed the homology of heavy chains among RBD antigen-specific B cell subtypes by BCR-seq analysis. Further single-cell BCR-seq analysis would be invaluable for synchronously comparing the homology of both heavy and light chains between IgA^+^ and IgG^+^ B cells, thus facilitating the identification of newly generated IgA^+^ B cell subtypes. The involvement of additional immune cells in the regulation of IgG-IgA class switching warrants further investigation. Our clinical study demonstrates that V-01 IN boost significantly augments the protective IgG and IgA in the serum of humans. However, comparisons were restricted to V-01 before- and after-boost responses due to a scarcity of volunteers for a comprehensive multiple-group analysis. Future investigations into the antigen-specific IgA^+^ B cells elicited by IN vaccination and antiviral protection in human trials will enhance our understanding and refine the IM+IN immunization approach.

In summary, we demonstrate that this IM+IN strategy can effectively induce a mucosal IgA response via direct class switching from IgG to IgA. Our findings highlight that the IFN-based vaccine does not require additional adjuvants to enhance mucosal immunity. Furthermore, this study provides valuable insights into the regulatory mechanisms of antigen-specific IgA production, thereby contributing to the advancement of effective mucosal vaccines.

## Methods

### Sex as a biological variable.

For animal models, both sexes of mice were examined. For clinical samples, both sexes were involved. The sex was not considered as a biological variable.

### Study design.

This study aimed to investigate the importance and mechanisms underlying the use of IN administration IPRF vaccine as a booster after IM priming. We assessed changes in immunity by comparing systemic and mucosal immune responses induced by IN versus IM boosts with the same IPRF fusion protein vaccine without adjuvants. Additionally, we evaluated the effectiveness and safety of this immunization strategy in clinical trials.

### Mice.

WT male and female C57BL/6J mice were obtained from SPF Biotechnology Co. Ltd. Age- and sex-matched mice were used for each experiment. Rag1^–/–^ mice were obtained from Gempharmatech Co. Ltd. Female k18-hACE2 mice were purchased from Gem Pharmatech Co. Ltd. WT and K18-hACE2 mice were used at 6–8 weeks old. Rag1^–/–^ mice were employed at 6–12 weeks old.

### Human participants and ethics.

An open-label, single-arm, single-center, investigator-initiated clinical trial was conducted to evaluate the safety and immunogenicity of the heterologous V-01 booster administered via nasal spray following prevaccination with inactivated COVID-19 vaccine CoronaVac. The V-01 vaccine developed by Livzon Mabpharm Inc. is the same as the mouse version IFN-Pan-RBD-Fc fusion protein molecular design with the mouse IFN-α replaced by human IFN-α. The low-dose group received 50 μg V-01, and the high-dose group received 100 μg V-01. The formulation contains 50 or 100 μg of fusion protein with 44.2 mg/mL trehalose and 0.2 mg/mL polysorbate 80 dissolved in 0.1 mL buffered saline.

Eligible individuals were healthy adults, aged 18 years or older, who had received 2 or more doses of CoronaVac in the preceding 6 months and had no COVID-19 or major diseases, including serious cardiovascular conditions and respiratory disorders such as asthma or chronic obstructive pulmonary disease. The key exclusion criteria included clinically confirmed or laboratory-confirmed COVID-19 or SARS-CoV-2 infection, nasal or oral diseases, known infection with HIV, and positive urine pregnancy test for women. Informed consent was obtained from all participants.

### Cell lines, viruses, and reagents.

293 F cells (Gibco) were cultured in SMM-TII medium (M293TII, Sino Biological) in Polycarbonate Erlenmeyer Flasks agitated at 135 rpm speed in an orbital shaker in an 8% CO_2_ incubator at 37 °C. The 293-ACE2 cell line, provided in-house. was cultured in Dulbecco’s modified Eagle’s medium (DMEM) supplemented with 10% heat-inactivated FBS, 100 U/mL penicillin, and 100 mg/mL streptomycin in a 5% CO_2_ incubator.

SARS-CoV-2 pseudovirus was generated in-house, as previously described. Briefly, human immunodeficiency virus backbones expressing firefly luciferase (pNL43R-E-luciferase) were cotransfected with pcDNA3.1 (Invitrogen) vectors encoding the SARS-VoV-2 S protein into 293 T cells (ATCC). Viral supernatants were collected 48 hours after transfection. Viral titers were quantified by luciferase activity using the Bright-Glo Luciferase Assay Vector System (Promega Biosciences).

### Protein expression and purification.

The COVID-19 vaccine protein was expressed in 293 F cells, as described previously. The coding sequence for the SARS-CoV-2 RBD (S protein amino acids 319–541, GenBank: YP_009724390) was codon optimized for mammalian cells and synthesized by GENewIZ, China. For IPRF expression, murine IFN-α4 was fused to the N-terminus of a CD4 helper epitope (PADRE, pan epitope) and RBD, with each component linked by a (G4S)4 linker. The IFN-α-pan-RBD sequence was then cloned into the PEE12.4 vector (Lonza) with a human IgG1 Fc, forming the IPRF fusion protein. The plasmid was transiently transfected into 293 F cells. The supernatant was harvested 7 days after transfection, and the protein was purified with a Protein A-Sepharose column (GE Healthcare) according to the manufacturer’s instructions. The purity and size of the protein were analyzed by SDS-PAGE.

### Mouse vaccination.

The fusion protein vaccine was diluted in PBS for administration. Mice were immunized intramuscularly with 10 μg IPRF in 100 μL using insulin syringes or were prepared for IN administration. Intranasal vaccination involved the full anesthesia of mice through an i.p. injection of a mixture comprising 200 μL 1.25% 2,2,2- Tribromoethanol and 2.5% Methyl-2-butanol. Subsequently, mice received nasal drops containing 5 μg of antigen in 5 μL per nostril. PBS was used as a control. Multiple samples were collected at the indicated time points to determine SARS-CoV-2 RBD-specific IgG, IgA, and neutralization antibody levels. The details of mouse vaccination were described in the figure legends.

### SARS-CoV-2 infection.

K18-hACE2 mice were challenged with SARS-CoV-2 (WIV04/2019) 16 days after the boost in the BSL-3 facility. Mice were lightly anesthetized with isoflurane and inoculated intranasally with 1 × 10^4^ FFU of SARS-CoV-2 viruses.

### Quantitative reverse transcription-polymerase chain reaction.

Viral RNA in lung tissues and nasal mucosa was determined by quantitative reverse transcription PCR (qRT-PCR). In brief, lung tissues and NTs were homogenized, and RNA was extracted with Trizol. A standard curve was generated by cloning the SARS-CoV-2 nucleocapsid (N) gene into a pcDNA3.1 expression plasmid, followed by in vitro transcription to obtain RNAs for standards. Viral RNA copies were quantified using Yeasen Hieff Unicon Universal TaqMan multiplex qPCR master mix with the following N-specific primers and probes: forward primer 5'-GGGGAACTTCTCCTGCTAGAAT-3', reverse primer 5'-CAGACATTTTGCTCTCAAGCTG-3', and probe 5'-FAM-TTGCTGCTGCTTGACAGATT-TAMRA-3'. Standard curves were obtained by 10-fold serial dilution of N standards, followed by qRT-PCR using the same 1-step SARS-CoV-2 RNA detection kit. The reactions were performed on a QuantStudio 6 Flex System (Applied Biosystems) under the following reaction conditions: 50°C for 15 minutes, 95°C for 30 seconds, and 45 cycles of 95°C for 10 seconds and 60°C for 30 seconds. The viral RNA copies of each tissue were expressed in copies per mL and represented as a log_10_ scale.

### Histopathology.

SARS-CoV-2–challenged K18-hACE2 mice were euthanized within the BSL-3 facility. Mouse lungs were harvested and fixed in 4% paraformaldehyde buffer for 48 hours, then embedded in paraffin. Sections (3–4 μm) were stained with H&E, and images were captured using a Pannoramic MIDI scanner.

### Extraction of mouse nasal mucosa supernatants.

Mice were anesthetized through an intraperitoneal injection of a solution composed of 400 μL of 1.25% tribromoethanol and 2.5% 2-butanol. Heart perfusion was performed using 100 mL of perfusion solution to eliminate peripheral blood. The lower jaw and tongue were excised using scissors. After detaching the scalp and any adjacent tissues, the heads were secured with pins on a wax dissection slab to expose the upper palate. The palates were carefully excised using a No. 11 scalpel blade, revealing the nasal-associated lymphoid tissue (NALT) adhered to the palate. A cut was made with scissors between the nasal and the frontal bones, isolating the anterior section, which includes the nasal mucosa along with the surrounding bone. This tissue was immersed in 1 mL of PBS and zirconium beads were added for homogenization. The mixture was then subjected to centrifugation at 13,887*g* (13,000 rpm) to obtain the supernatant.

### ELISA.

The 96-well plates (Corning) were coated with 100 μL SARS-Cov-2 RBD (1.5 μg/mL, SPE-C52H3, Acro Biosystems) overnight at 4 °C. Following PBS washes, the plates were blocked with a blocking buffer (PBS containing 5% skim milk powder) the next day. Immunized animal serum samples, nasal washes, BALF, or nasal mucosal supernatants were serially diluted and added to the blocked plates, followed by incubation at 37 °C for 1 hour. Subsequently, the plates were washed with PBST (PBS containing 0.05% Tween 20) and incubated with goat anti-mouse IgG-HRP (1:5,000, Cwbiotech, CW0102S) at 37 °C for 30 minutes or goat anti-mouse-IgA-HRP (1:2,000, Abcam, ab97235) for 45 minutes. After further PBST washes, the HRP substrate TMB was added. The reactions were stopped by 2 M sulfuric acid, and the absorbance at 450–630 nm was read using a microplate reader (Molecular Devices). The endpoint titers were defined as the reciprocal of summing the average OD value of the lowest dilution of mouse serum in the PBS group and 3-fold of its SD. The anti-RBD IgG and IgA levels in human plasma samples were measured by ELISA Kit (IgG, DD3112-P, IgA, DD3108, Vazyme Biotech).

### Pseudovirus neutralization assay.

The pseudovirus was produced by cotransfection of the plasmids expressing firefly luciferase (pNL43R-E-luciferase) and pcDNA3.1 expressing the SARS-CoV-2 S protein into 293 T cells. After 48 hours, the viral supernatant was collected, and viral titers were determined by luciferase activity in relative light units. To evaluate the neutralizing activity of vaccinated mice or human serum, 293-hACE2 cells were seeded into 96-well plates (1 × 10^4^ per well). Heat-inactivated serum samples were serially diluted threefold and incubated with 50% of the tissue culture infectious dose (TCID_50_) of pseudovirus for 1 hour at 37 °C. The medium containing pseudovirus alone was used as a control. Following incubation for 24 hours, the luciferase substrate was added according to the manufacturer’s instructions, and luciferase activity was determined by the Bright-LiteTM Luciferase Assay System (Vazyme). The 50% neutralizing titer (NT_50_) was defined as the reciprocal of serum dilution at which the relative light units (RLU) were reduced by 50% compared with virus control wells.

### SARS-CoV-2 virus neutralization assay.

The focus reduction neutralization test (FRNT) was utilized to evaluate the serum neutralization effect. The serum samples from immunized K18-hACE2 mice were inactivated at 56 °C for 30 minutes and then serially diluted at a 1:3 ratio with cell culture medium. The diluted sera were coincubated with 200 FFU WT SARS-CoV-2 strain at 37°C for 1 hour. Subsequently, the mixtures were transferred to preseeded Vero E6 cell plates. After 1 hour of infection, the mixtures were removed and cell plates were overlaid with Minimum Essential Medium (MEM) containing 1.2% Carboxymethylcellulose (CMC) for 24 hours of culture. Cell plates were then fixed with 4% paraformaldehyde followed by virus focus staining utilizing SARS-CoV/SARS-CoV-2 Nucleocapsid Rabbit PAb (Sino Biological, 40143-T62) and Peroxidase AffiniPure Goat Anti-Rabbit IgG (H+L) (Jackson, 111-035-144). Virus foci were visualized using KPL TrueBlue Peroxidase substrate (Seracare Life Science, 5510–0030) and analyzed with the CTL ImmunoSpotS6 Ultra analyzer (Cellular Technology Limited). The FRNT_50_ was defined as the serum dilution at which neutralization antibodies inhibited 50% of the viral infection.

### SARS-CoV-2 RBD B cell tetramer preparation and staining.

Recombinant SARS-CoV-2 spike RBD His Biotin Protein (SPE-C52H3, Acro Biosystems) was incubated at a 4:1 molar ratio with either streptavidin-PE (Biolegend), streptavidin-APC (Biolegend), or streptavidin-APC-Cy7 (Biolegend) for 30 minutes at 4°C. The resulting mixture was then purified, concentrated using an Amicon Ultra (50 kDa MWCO) spin column, and washed with cold, sterile PBS.

### Cell isolation from mouse model and flow cytometry.

Mononuclear cells from nasal and lung tissues were isolated using an enzymatic digestion method. Briefly, lungs and NTs were minced and incubated in a digestion cocktail containing collagenase A and DNase I in FACS buffer at 37°C for 30 minutes, followed by dissociation through a 70-mm filter. Cells from the interface were collected, and the erythrocytes were lysed with ammonium-chloridepotassium (ACK) buffer. Splenocytes were similarly collected and treated with ACK buffer for erythrocyte lysis. Mandibular and MLNs and NALT were minced and processed as described for lung tissues, followed by single-cell collection.

For flow cytometric analysis, single-cell suspensions were prepared in FACS buffer, blocked with anti-CD16/32 (anti-FcγIII/II receptor, clone 2.4G_2_), and stained with specific fluorescence-labeled antibodies. To sort antigen-specific B cells from immunized mice, cells were incubated with anti-CD45-AF700 (30-F11, Biolegend), anti-B220-BV650 (RA3-6B2, Biolegend), anti-CD3-PE-Cy7 (145-2C11, Biolegend), and 2 SARS-CoV-2 RBD tetramer for 30 minutes at 4°C. To sort B cells for transfer to Rag-1 mice, cells were incubated with anti-CD45 AF700 (30-F11, Biolegend), anti-B220-BV650 (RA3-6B2, Biolegend), anti-IgM-PE-Cy7 (RMM-1, Biolegend), anti-IgD-PerCP-Cy5.5 (11-26c.2a, Biolegend), anti-IgG-PE (polyclone, SouthernBiotech), anti-CD3-ef450 (17A2, eBioscience), and anti-CD4-FITC (GK1.5, Biolegend) for 30 minutes at 4°C. For intracellular IgA staining, mouse splenocytes or lymphocytes from lung and nasal tissues were seeded in U-bottom 96-well plates (1 × 10^6^/well) and stimulated with a 5 μg/mL SARS-CoV-2 RBD protein and 5 μg/mL Brefeldin A (Biolegend) for 6 hours. Cells were then incubated with anti-CD45 AF700 (30-F11, Biolegend), anti-B220-BV650 (RA3-6B2, Biolegend), anti-CD38-APC (90, Biolegend), anti-CD138-BV711(281-2, Biolegend), anti-GL-7-ef450(GL7, eBioscience), anti-IgM-PE-Cy7 (RMM-1, Biolegend), anti-IgD-PerCP-Cy5.5 (11-26c.2a, Biolegend), and anti-IgA (polyclone, unlabeled, SouthernBiotech). After fixation and permeabilization, intracellular IgA was labeled using anti-IgA-FITC (polyclone, SouthernBiotech) Flow cytometry data were acquired on a BD Fortessa Flow Cytometer and analyzed using FlowJo Software (10.5.3; Tree Star).

### ELISPOT assay.

Murine IFN-γ ELISPOT assays were performed following the manufacturer’s protocols for the mouse IFN-γ ELISPOT kit (BD Bioscience). Splenocytes and lymphocytes from lymph nodes of immunized mice were seeded in the plates at a density of 5 × 10^5^ cells per well. The nasal and lung lymphocytes were seeded at a density of 2 × 10^5^ cells per well. Cells were incubated with a peptide pool of 15-mer peptides overlapping by 11 amino acids for SARS-CoV-2 RBD protein (5 μg/mL) in precoated 96-well ELISPOT plates (BD Biosciences) with anti-mouse IFN-γ at 4°C overnight. Concanavalin A (ConA, Sigma-Aldrich) was used as a positive control and medium was used as a negative control. Following cell removal, a biotinylated anti-mouse IFN-γ detection antibody (BD Bioscience) was added and plates were incubated for 2 hours at room temperature. The plates were washed 3 times with PBST before adding Streptavidin-HRP (BD Bioscience). Spots were developed using BD ELISPOT AEC substrate (BD Bioscience) and counted with an automated ELOSPOT reader (Cellular Technology). For the B cell ELISPOT assay, splenocytes and nasal and lung lymphocytes from immunized mice were seeded in plates coated with 5 μg/mL RBD protein. After 16 hours, cells were removed, and biotinylated goat anti-mouse IgG or anti-mouse IgA (Abcam) was added into plates followed by incubation for 2 hours at room temperature. The plates were washed 3 times with PBST, and Streptavidin-HRP (BD Bioscience) was added. Spots were developed with BD ELISPOT AEC substrate (BD Bioscience), counted, and analyzed using an automated ELOSPOT reader (Cellular Technology).

### RBD-specific B cell sorting and BCR sequencing.

Seven days after IN or IM boost immunization in the mouse model, single-cell suspensions from mandibular lymph nodes (IM+IN) or inguinal lymph nodes (IM+IM) were prepared as described above. The RBD^+^B220^+^ B cells were sorted as outlined above. Total RNAs were extracted using the MagMAX mirVana Total RNA Isolation Kit (A27828, Thermo Fisher Scientific). 2 micrograms of RNA from each sample were used to prepare the BCR library with KC-Digital Stranded BCR-seq Library Prep Kit for Illumina (DT0811-02, SeqHealth). Duplication bias was depleted using an unique molecular identifier (UMI) of 8 random bases to tag preamplified cDNA. The NovaSeq (Illumina) was employed for sequencing library products at 250–500 bp. Raw data were filtered using fastp (version 0.23.0), and low-quality reads were discarded. The kcUID (SeqHealth internal UID processing software) was used to merge the reads with similar UIDs for error correction and removal of redundant reads. Consensus sequences were extracted and subjected to BCR-seq analysis. Reads were mapped to the international ImMunoGeneTics (IMGT) database using MiXCR software (v.3.0.3) to obtain V and J gene fragments as well as the CDR3 sequences. The mutation rate of IGHV was quantified as the number of mutated bases per 10 kb. BCR clones were quantified by clustering IGH sequences that utilized the same V/J alleles and exhibited less-than or equal-to 1 mutation in the CDR3 region. Raw BCR sequencing data for all mice are available on Sequence Read Archive (SRA) under BioProject: PRJNA1165948.

### Statistics.

All statistical analyses were performed using GraphPad Prism 9.5. Data are presented as the mean ± SEM. Statistical significance between the 2 groups was assessed using an unpaired Student’s 2-tailed *t* test. Differences among multiple groups were evaluated using 1-way ANOVA with Tukey’s multiple comparison test or 2-way ANOVA with Tukey’s multiple comparisons test. *P* values of < 0.05 were considered significant. Data were analyzed using GraphPad Prism v9.5.0.

### Study approval.

Animal care and experimental procedures adhered to institutional protocol and guidelines, with approval (SYXK2021122) from the Animal Care and Use Committee of the Institute of Biophysics, Chinese Academy of Sciences. For human clinical trial, informed consent was obtained from all participants. The study protocol of human was approved by the Ethics Committee of Shenzhen Third People’s Hospital (IRB202206702).

### Data availability.

All data associated with this study are present in the paper or the Supplemental Materials. Raw BCR sequencing data for all mice are available on NCBI Sequence Read Archive (SRA) database (https://submit.ncbi.nlm.nih.gov/subs/sra/) under BioProject: PRJNA1165948. Source data for this work are provided in the Supporting Data Values file.

## Author contributions

YXF, HP, and YL conceptualized the project. YL, XL, XC, Z Zhang, XW, XH, HL, ZR, ZH, and JY developed the methodology. YXF, HP, Zhaoyong Zhang, JZ, YL, XL, XC, Zheng Zhang, XW, XH, BJ, FQ, HX, and YW conducted the investigation. YL, XL, XC, and Z Zhang were responsible for visualization. HP and Zheng Zhang acquired funding. HP and YXF were the project administrators. YXF and HP supervised the project. YL, HP, and YXF wrote the original draft of the manuscript. YXF, HP, YL, Zheng Zhang, and JZ reviewed and edited the manuscript.

## Supplementary Material

Supplemental data

Supporting data values

## Version 1 01/14/2025

In-Press Preview

## Version 2 03/03/2025

Electronic publication
