# Supplementary material for: Sequential intranasal booster triggers class switching from intramuscularly primed IgG to mucosal IgA against SARS-CoV-2
Source: J Clin Invest. 2025 Jan 14;135(5):e175233. doi: 10.1172/JCI175233 (PMC11870729; doi:10.1172/JCI175233)
Supplement: Supplemental data [file jci-135-175233-s037.pdf]

Supplemental Materials

Supplemental Figure 1. IN sequential immunization induces comparable IgG response to IM boosting.

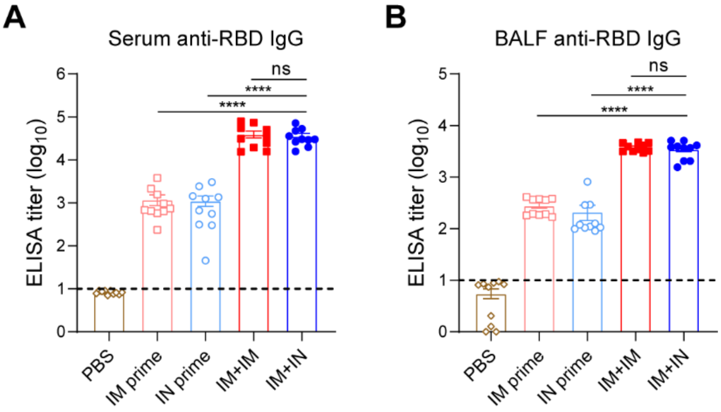

C57BL/6 mice (n=10) were primed with 10μg IPRF via IM and boosted via IM or IN routes. (**A** and **B**) The RBD-specific IgG antibody responses in serum and BALF of immunized mice were evaluated 28 days post-priming by ELISA. The dotted lines represent the endpoint of these ELISA tests. Data are presented as mean ± SEM. P values were determined by one-way ANOVA with Tukey's multiple comparisons test; ns (not significant), ****$P < 0.0001$. Individual data points are represented and were pooled from 2 independent experiments.

Supplemental Figure 2. IN sequential immunization induces comparable resident IgG+ B cell response to IM boosting.

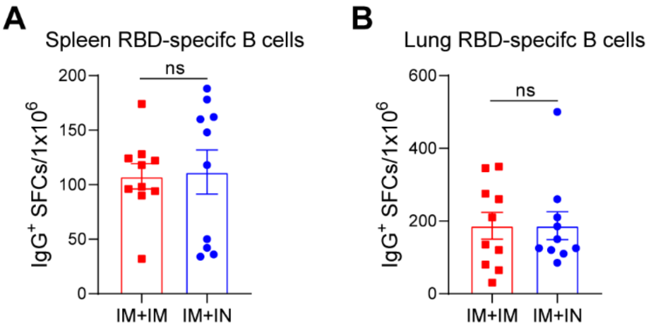

C57BL/6 mice (n=10) were primed with 10 μg IPRF via i.m. and boosted via i.m. or i.n. routes. Spleen and lung tissue were collected on day 14 after boosting. (**A** and **B**) ELISPOT assays evaluated IgG+ B cells from mouse splenocytes, and lung lymphocytes cocultured with RBD protein. Data are presented as mean ± SEM. P-values were determined by unpaired t-tests; ns (not significant). Individual data points are represented and were pooled from 2 independent experiments.

Supplemental Figure 3. IN sequential immunization induces robust systemic and mucosal IgA$^+$ B cell response determined by intracellular staining.

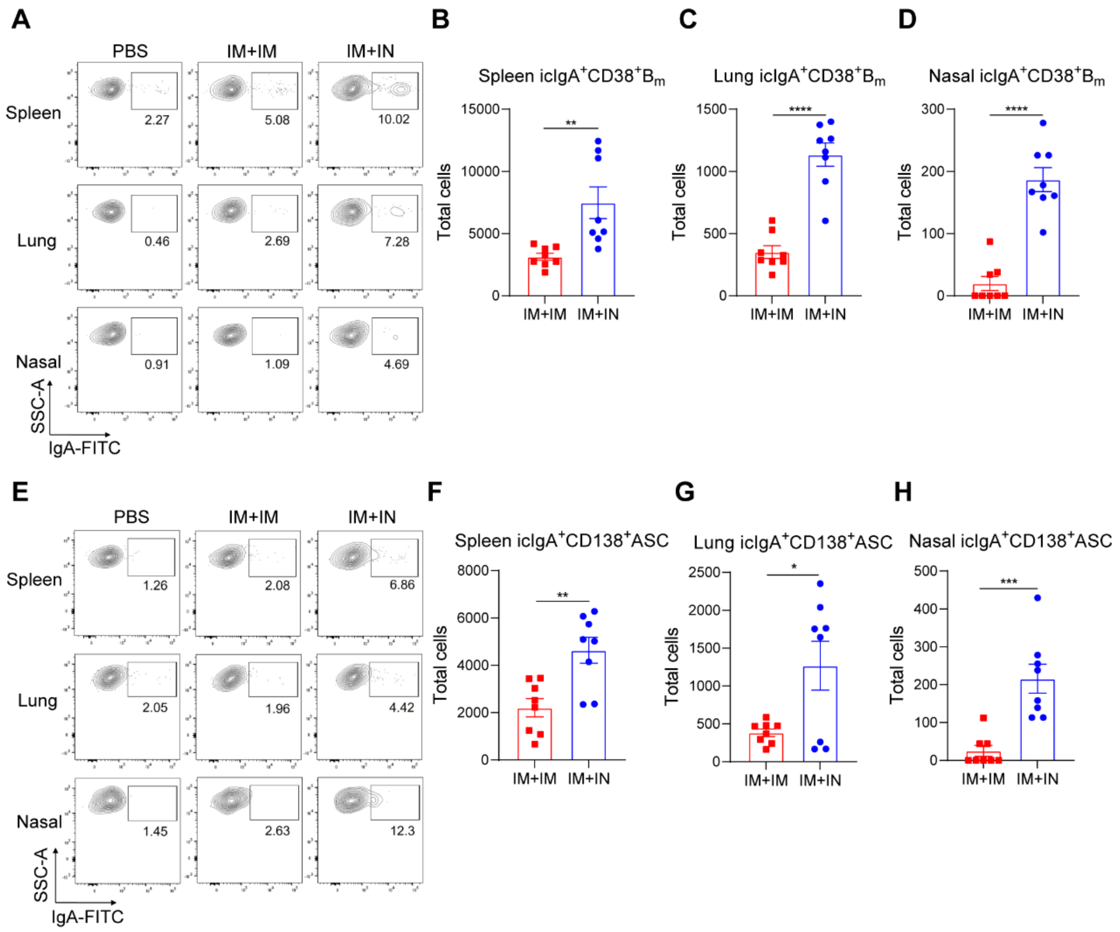

C57BL/6 mice (n=8) were primed with 10μg IPRF via i.m. and boosted via i.m. or i.n. routes. Spleen, lung, and nasal tissues were collected on day 14 post-boosting. (**A-H**) Intracellular antibody detection was performed for IgA$^+$ B_m cells (icIgA$^+$CD38$^+$GL-7$^-$) and IgA$^+$ ASCs (icIgA$^+$CD138$^+$) from splenocytes, lung, and nasal lymphocytes cocultured with RBD protein. Representative flow cytometric contour plots are shown in (**A**) and (**E**). (**B-D**, **F-H**) Total IgA$^+$ B_m cells and IgA$^+$ ASCs from spleen, lung, and nasal tissue are reported. Data are presented as mean ± SEM. P-values were determined by unpaired t-tests; *P<0.05, **P<0.01, ***P<0.001, ****P<0.0001. Individual data points are represented and were pooled from 2 independent experiments.

Supplemental Figure 4. The RBD-specific antibody response induced by sequential IM+IM or IM+IN vaccination in K18-hACE2 mice.

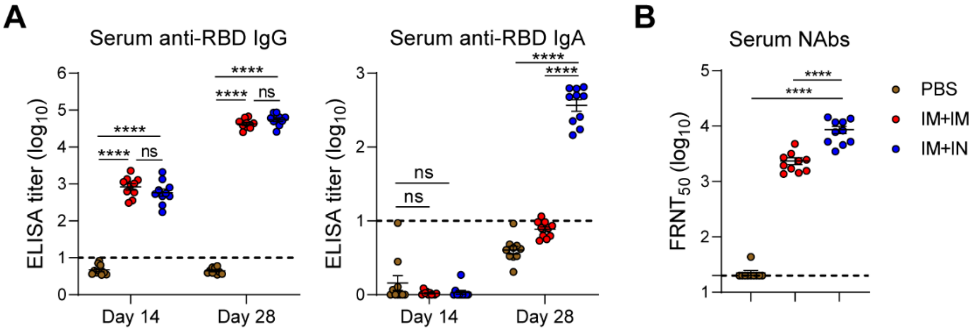

The K18-hACE2 transgene mice (n=10) were immunized intramuscularly with 10μg IPRF or PBS on days 0 and boosted with 10μg IPRF via i.m. or i.n. route. (**A**) RBD-specific IgG and IgA antibody responses in serum of immunized mice were evaluated at 14 and 28 days after priming by ELISA. The dotted lines represent the endpoint of these ELISA tests. (**B**) The neutralization activity of vaccinated sera collected on day 28 was evaluated using a SARS-CoV-2 virus neutralization assay. The dotted lines represent the minimum dilution. Data are presented as mean ± SEM. P-values were determined by one-way ANOVA with Tukey's multiple comparisons test. ns (not significant), ****P<0.0001.

Supplemental Figure 5. The RBD-specific IgG responses via various administration routes

A

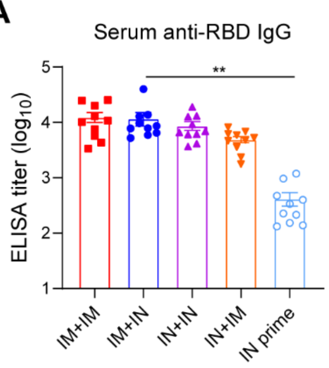

C57BL/6 mice (n=10) were primed with 10μg IPRF via either intramuscular or intranasal routes and subsequently boosted via either route. **(A)** RBD-specific IgG antibody levels in serum were evaluated 7 days post-boost by ELISA. Data are presented as mean ± SEM. P values were determined by one-way ANOVA with Tukey's multiple comparisons test; **P<0.01. Individual data points are represented and were pooled from 2 independent experiments.

Supplemental Figure 6. Only IM+IN induces IgA sequence which shown comparable CDR3 length and V-J gene usage with IgG.

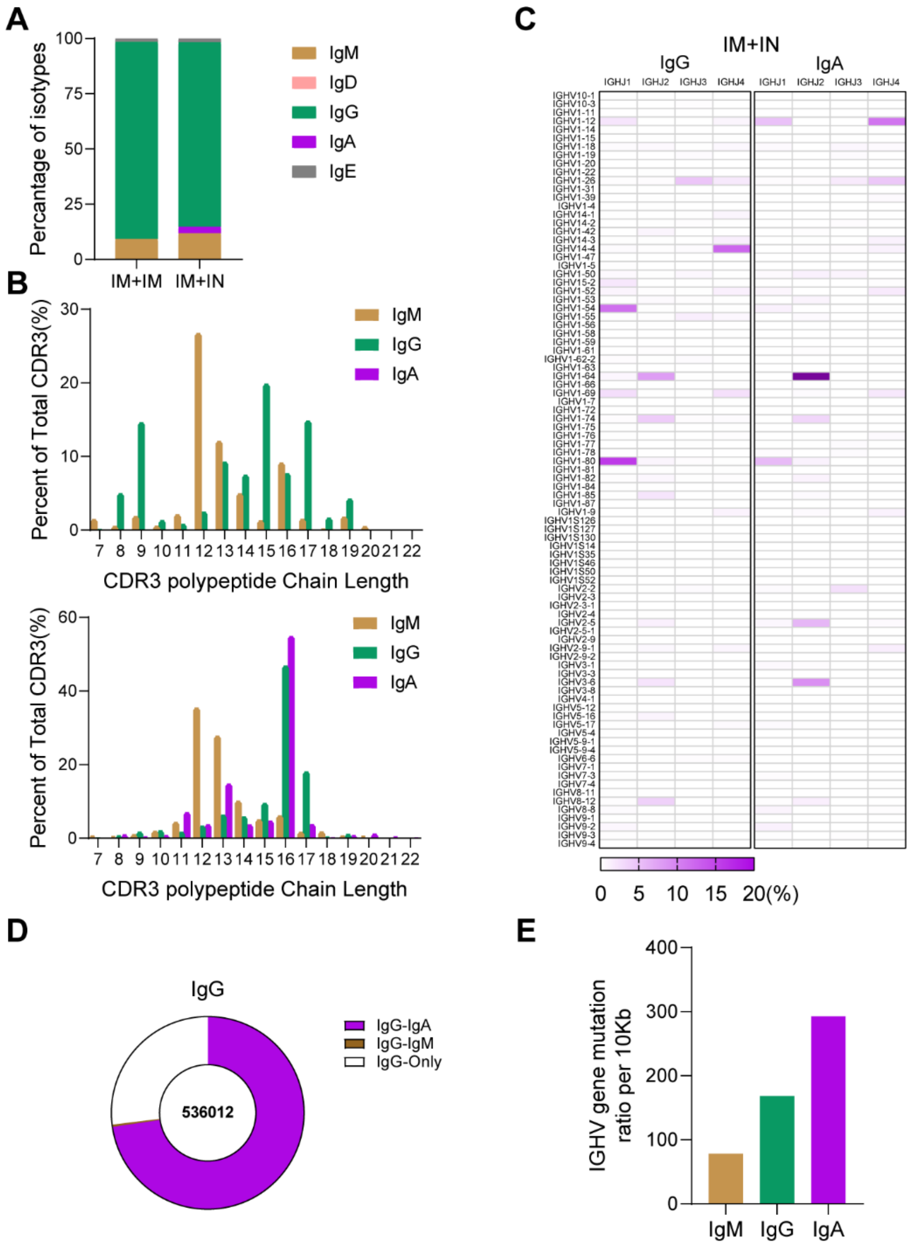

(**A**) The percentage distribution of isotypes in the IM+IM and IM+IN group. (**B**) The percentage of CDR3 length distribution for each isotype in IM+IM and IM+IN groups. (**C**) V-J gene pair usage in the IM+IN groups. (**D**) The ratio of the identical clone type of IgG is shown in a pie chart. (**E**) IGHV and IGHJ gene mutation rates per 10Kb in IM+IM and IM+IN groups.

Supplemental Figure 7. The RBD-specific IgA response following an IN boost with or without CD4+ T cell depletion.

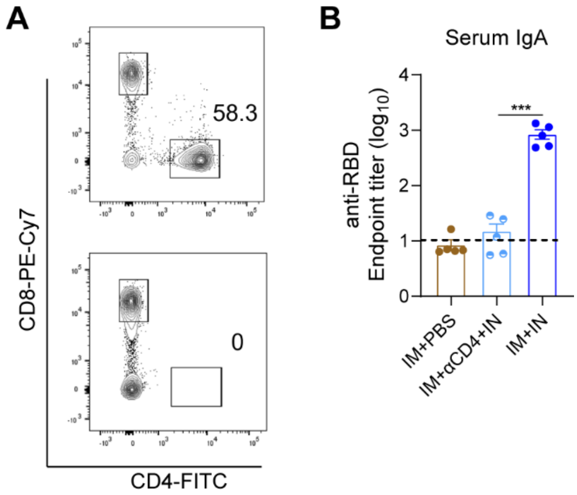

C57BL/6 mice (n=5) were primed with 10μg IPRF via intramuscular administration, and 14 days later, $CD4^+$ T cells were deleted by anti-mouse CD4 antibody via intravenous injection. Mice were then intranasally immunized with 10 μg IPRF or PBS. (**A**) Flow cytometry analysis demonstrating the efficiency of $CD4^+$ T cell depletion. (**B**) RBD-specific IgA antibody responses in serum were evaluated 14 days post-boost by ELISA. The dotted lines represent the endpoint of these ELISA tests. Data are presented as mean ± SEM. P-values were determined by one-way ANOVA with Tukey's multiple comparisons test; **P<0.01

Supplemental Figure 8. RBD-specific IgG response induced by IM or IN boosts in Rag-1 mice that were transferred with IgG⁺ B cells and CD4⁺ T cells.

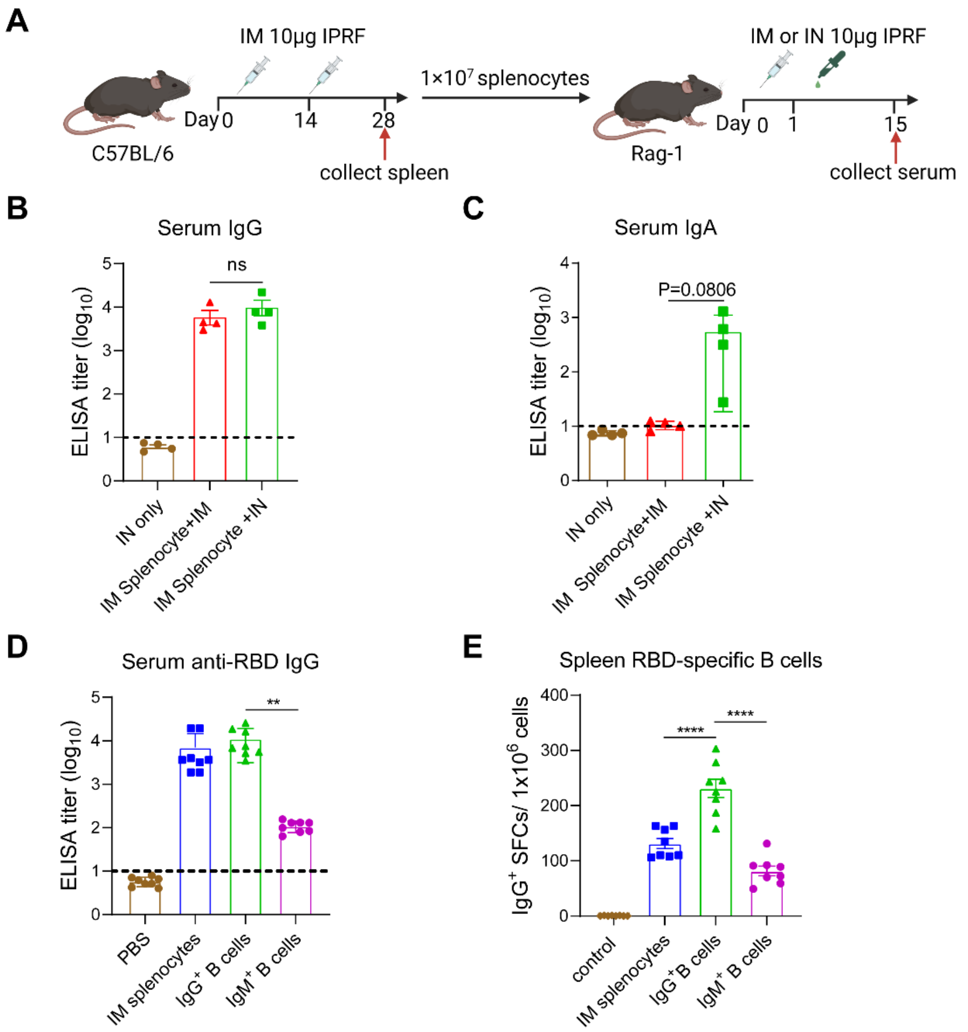

(**A**) Splenocytes from C57BL/6 mice (n=10) receiving two doses of intramuscular vaccination were collected and adoptively transferred into Rag-1 mice (n=4) one day before intranasal or intramuscular administration of the 10 μg IPRF. (**B and C**) RBD-specific IgG and IgA antibody responses in the serum of immunized Rag-1 mice were evaluated on day 14 post-vaccination by ELISA. (**D and E**) Splenocytes from C57BL/6 mice (n=10) receiving two doses of i.m. vaccination were collected and sorted into IgG$^+$ and IgM$^+$ B cells, and CD4$^+$ T cells, which were then adoptively transferred into Rag-1 (n=8) mice one day before intranasal administration of 10 μg IPRF. (**D**) RBD-specific IgG antibody responses in the serum of immunized Rag-1 mice were evaluated 14 days

after vaccination by ELISA. (**E**) ELISPOT assays measured IgG^+ B cells from splenocytes cocultured with RBD protein. Data are presented as mean ± SEM. P-values were determined by one-way ANOVA with Tukey's multiple comparisons test; ns (not significant), **P<0.01, ****P<0.0001. Individual data points are represented and pooled from two independent experiments.

Supplemental Figure 9. Gating strategies for B cells.

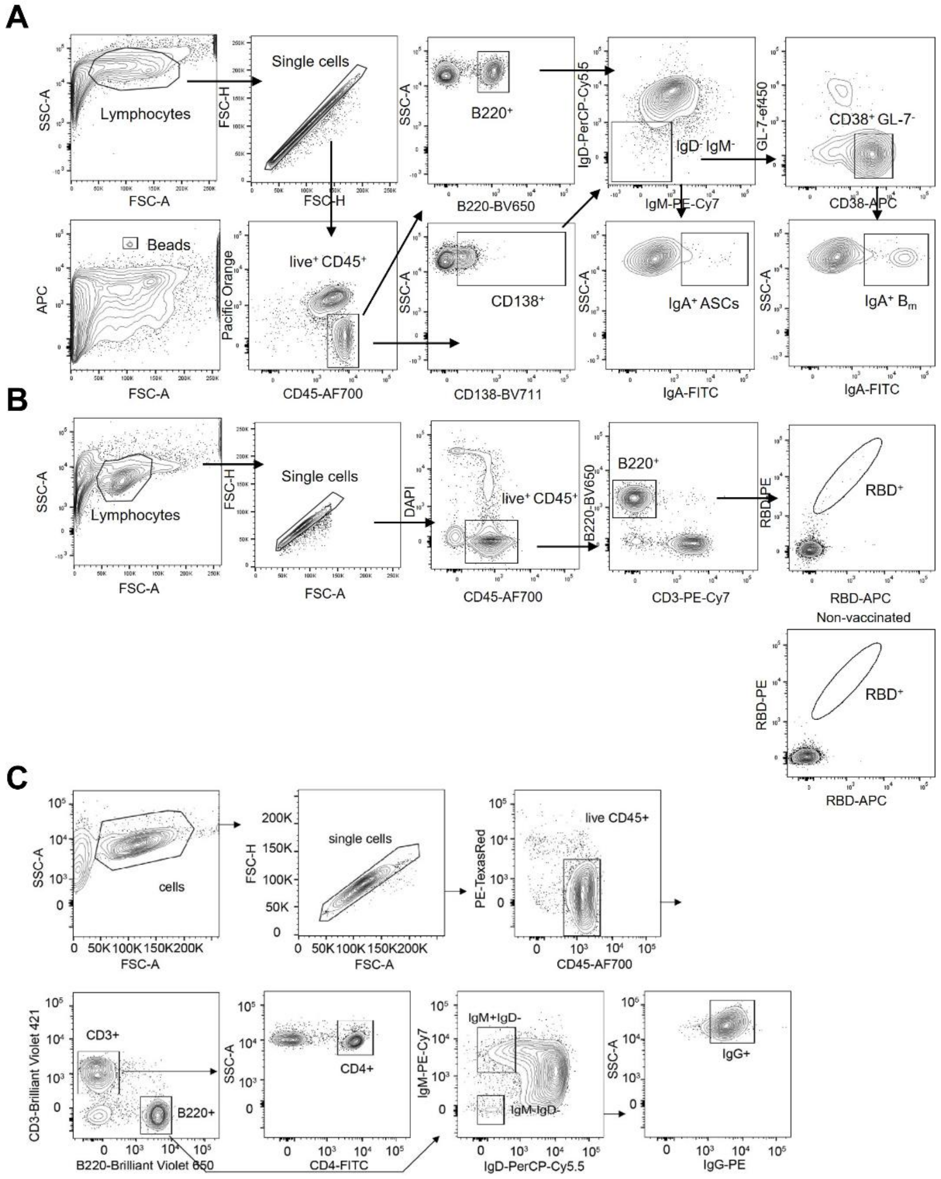

(**A**) Gating strategy of intracellular staining of IgA$^+$ B$_m$ and IgA$^+$ ASCs (**B**) Gating strategy to sort RBD-specific B cells from DLN after IM+IN immunization. (**C**) Gating strategy for sorting CD4$^+$ T cells, IgM$^+$, and IgG$^+$ B cells from solenocytes following IM+IM immunization.

Supplemental Table 1.

Table 1 Demographic characteristics of participants receiving nasal spray vaccine.

Characteristics	All patients (n=30)	Low-dose Nasal spray vaccine(n=5)	High-dose Nasal spray vaccine(n=25)	*P* value
Age (years), mean±SD (n)	41.21±10.83	31.6±6.43	42.88±9.78	0.020
Sex, male, n (%)	7(23.3)	1(20.0)	6(24.0)	1.000
Basic diseases, n (%)	6(20.0)	1(20.0)	5(20.0)	1.000
COVID-19 vaccines, n (%)				
Two	13(43.3)	3(60.0)	10(40.0)	0.628
Three or more	17(56.7)	2(40.0)	15(60.0)	0.628

Supplemental Table 2.

Table 2 Solicited and unsolicited adverse reactions that occurred within 14 days after booster vaccination.

		Number of adverse reactions			
		Low-dose Nasal spray vaccine (n =5)	High-dose Nasal spray vaccine (n = 25)	Total (n=30)	*P* value
Solicited adverse reactions within 14 days					
	Any	1(20%)	4(16%)	5(16.7%)	0.627
	Grade 3	0(0%)	0(0%)	0(0%)	
Local adverse reactions					
Total	Any	1(20%)	2(8%)	3(10%)	0.433
Dry nose	Any	1(20%)	1(4%)	2(6.7%)	0.310
Oral mucositis	Any	0(0%)	0(0%)	0(0%)	
Pharyngeal swelling	Any	1(20%)	0(0%)	1(3.3%)	0.167
Nasal congestion	Any	1(20%)	0(0%)	1(3.3%)	0.167
Sneeze	Any	0(0%)	1(4%)	1(3.3%)	1.000
Runny nose	Any	0(0%)	1(4%)	1(3.3%)	1.000
Hoarseness	Any	0(0%)	0(0%)	0(0%)	
Systemic adverse reactions					
Total	Any	1(20%)	1(4%)	2(6.7%)	0.310
Nausea	Any	0(0%)	0(0%)	0(0%)	
Fever	Any	0(0%)	0(0%)	0(0%)	
	Grade 3				
Diarrhe	Any	0(0%)	0(0%)	0(0%)	
Arthralgia	Any	0(0%)	0(0%)	0(0%)	
Myalgia	Any	0(0%)	1(4%)	1(3.3%)	1.000
Cough	Any	0(0%)	0(0%)	0(0%)	
Oropharyngeal pain	Any	1(20%)	0(0%)	0(0%)	0.167
Appetite impaired	Any	0(0%)	0(0%)	0(0%)	
Fatigue	Any	1(20%)	0(0%)	1(3.3%)	0.167

Headache	Any	0(0%)	0(0%)	0(0%)	
Dyspnoea	Any	0(0%)	0(0%)	0(0%)	
Arthralgia	Any	0(0%)	0(0%)	0(0%)	
	Grade 3				
Pruritus	Any	0(0%)	0(0%)	0(0%)	
Unsolicited adverse reactions within 14 days					
Total	Any	0(0%)	1(4%)	1(3.3%)	1.000
Insomnia	Any	0(0%)	1(4%)	1(3.3%)	1.000

Any= all the participants with any adverse reactions.
